# Barriers and facilitators to representative acute leukemia trial participation at National Cancer Institute-designated comprehensive cancer centers

**DOI:** 10.1093/oncolo/oyaf241

**Published:** 2025-08-08

**Authors:** Andrew Hantel, Eric C Blackstone, Anna C Revette, Jane E Roberts, Alice S Mims, Daniel J DeAngelo, Christopher S Lathan, Gregory A Abel

**Affiliations:** Department of Medical Oncology, Dana-Farber Cancer Institute, Boston, MA 02215, United States; Harvard Medical School, Boston, MA 02115, United States; HMS Center for Bioethics, Boston, MA 02115, United States; Department of Medical Oncology, Dana-Farber Cancer Institute, Boston, MA 02215, United States; HMS Center for Bioethics, Boston, MA 02115, United States; Department of Medical Oncology, Dana-Farber Cancer Institute, Boston, MA 02215, United States; Department of Medical Oncology, Dana-Farber Cancer Institute, Boston, MA 02215, United States; The Ohio State University, Columbus, OH 43210, United States; Department of Medical Oncology, Dana-Farber Cancer Institute, Boston, MA 02215, United States; Harvard Medical School, Boston, MA 02115, United States; Department of Medical Oncology, Dana-Farber Cancer Institute, Boston, MA 02215, United States; Harvard Medical School, Boston, MA 02115, United States; Department of Medical Oncology, Dana-Farber Cancer Institute, Boston, MA 02215, United States; Harvard Medical School, Boston, MA 02115, United States; HMS Center for Bioethics, Boston, MA 02115, United States

**Keywords:** acute leukemia, clinical trial enrollment, health equity, health disparities

## Abstract

**Background:**

Demographic enrollment disparities in adult acute leukemia clinical trials limit generalizability and diminish opportunities for minoritized populations to benefit from novel therapies. The distinct natural history and clinical research landscape of acute leukemia, the latter of which is centralized at the National Cancer Institute-designated Comprehensive Cancer Centers (CCCs), pose challenges to eliminating this disparity.

**Materials and Methods:**

We performed an analysis of 2 qualitative focus group and interview-based studies to characterize barriers and facilitators to representative acute leukemia clinical trial participation. Participants were patients with acute leukemia belonging to under-enrolled demographic groups (female, older adults, Hispanic ethnicity, or non-White race) and clinicians who treat and enroll them in clinical trials at CCCs in Massachusetts and Ohio.

**Results:**

Eight focus groups and 7 individual interviews were performed with 61 participants (34 patients and 27 clinicians). Barriers idiosyncratic to acute leukemia included inpatient and resource-intensive outpatient care, limited time between diagnosis and treatment initiation, low disease prevalence leading to trial centralization, and barriers related to hematopoietic stem cell transplantation. Barriers common across cancer types included restrictive eligibility criteria, complex study logistics, and cost. Potential solutions included local hospital and CCC partnerships and navigation to enhance timely referral and co-management during participation, multi-modal trial education and peer support in the peri-diagnostic period, minimizing unnecessary eligibility criteria, and travel and financial assistance.

**Conclusions:**

Patients and clinicians identified actionable steps to improve trial participation that address the unique challenges for adults with acute leukemia. A multi-pronged and disease-specific approach is needed to achieve representative acute leukemia clinical trials.

Implications for practice This qualitative analysis of focus groups and interviews with acute leukemia clinicians and historically under-enrolled patient populations identified disease-specific factors that limit representative clinical trial enrollment at NCI-designated Comprehensive Cancer Centers and highlighted actionable solutions. Such solutions include minimizing trial eligibility criteria, decentralizing trial care through referral site collaboration for tests and imaging, leveraging peers and community partners for peri-diagnostic education, and enhancing financial and travel assistance, especially for caregivers during long hospitalizations and surrounding transplantation. Implementing these strategies is necessary to ensure that minoritized groups can representatively participate in and benefit from acute leukemia trials.

## Introduction

Demographic disparities in cancer clinical trial participation bias research findings, limit generalizability, and reduce equitable access to the novel therapies and high-quality care that trials provide.^1–3^ Acute myeloid and lymphoblastic leukemia are aggressive blood cancers that affect 190 000 persons nationally^4^ as of 2021 and generated 278 new trials in 2023.^5^ The incidence-adjusted enrollment of self-identified Black and/or Hispanic patients in adult acute leukemia trials is among the lowest known—up to 85% lower than White patients.^6–8^ Older adults, female patients, and patients residing in neighborhoods with a high area deprivation index have also been shown to be underrepresented in acute leukemia research.^8–11^ These leukemias have unique care patterns that limit the utility of existing outpatient- or community-focused enrollment disparity interventions.^3^^,^^12–16^ Given that patients require rapid inpatient therapy, up to half are cared for at Comprehensive Cancer Centers (CCCs), and hematopoietic stem cell transplantation (HSCT) is often required.^17–19^

Our prior analysis of multi-institutional clinical trials from the Cancer and Leukemia Group B, now the Alliance for Clinical Trials in Oncology, found that participatory disparities by race and ethnicity have been propagated by inequitable enrollment at CCCs relative to the catchment areas for which they are responsible.^8^ Qualitative and quantitative studies have documented numerous enrollment barriers and facilitators to cancer clinical trial enrollment at the individual, interpersonal, institutional, and societal levels, as outlined in the National Institute for Minority Health and Health Disparities (NIMHD) research framework.^3^^,^^13–16^ In a recent study, we found several well-documented reasons for disparities in clinical trial enrollment at CCCs, but that post-access factors may be more contributory to the overall disparity in enrollment.^20^ Despite the unique care patterns of adult acute leukemia and recent advances in the therapeutic landscape, few studies have included this patient population, limiting assessment of which obstacles are most pressing and how they might be addressed within current care patterns unique to these conditions.

To address this knowledge gap, we sought to characterize stakeholder-identified barriers and facilitators to representative adult acute leukemia clinical participation at CCCs. We also sought to identify factors contributing to post-access enrollment inequities that were unexplained in previous analysis.^20^ We report the results of a secondary qualitative analysis that blends 2 aligned studies characterizing these factors, identifying targets for complementary quantitative research and future interventions.

## Materials and methods

### Theoretical framework, study design, and objectives

The NIMHD research framework^21^ served as the theoretical framework upon which our analysis was based. This framework conceptualizes factors relevant to disparities by their *levels of influence*, from the individual to the societal, and their *domains of influence*, from the biological to the health care system. This framework promotes a holistic approach to exploring, identifying, assessing, and intervening on health disparities.

This analysis integrated 2 qualitative studies with aligned research objectives and participant types that were performed in series. The first study’s objective was to explore barriers and facilitators to participation in acute leukemia research participation, as defined by patients from under-enrolled groups and their clinicians. The second study sought to characterize how acute leukemia-specific barriers and facilitators to research participation might interact with interventions and/or policy developments; this study had the same participant types as the first. Accordingly, a constructivist paradigm was followed, which led to the use of a qualitative approach. Both focus groups and individual interviews were performed to enable idea sharing between patients and between healthcare professionals and to allow those who were not comfortable in a group setting to participate. Both studies assessed barriers and facilitators across NIMHD domains and levels. The second study also sought input on the construction and revision of a specific behavioral intervention; these data are not included in this analysis. Based on the alignment of these studies’ objectives, design, and context, the primary objective of this analysis was to characterize barriers and facilitators to representative acute leukemia clinical participation at CCCs.

### Participants and recruitment

There were 2 types of participants in the studies: patients and leukemia clinicians. Patients were adults diagnosed with acute leukemia who self-identified from one or more previously identified under-enrolled groups (female, older adult, Hispanic ethnicity and/or race other than White, or who resided in a neighborhood with a high area deprivation index score),^8^ and who had received leukemia care at Dana-Farber Cancer Institute/Brigham and Women’s Hospital, Massachusetts General Hospital, or Beth Israel Deaconess Medical Center. Clinicians were physicians, nurse practitioners, and physician assistants who cared for patients with acute leukemia at these sites and who enrolled them in clinical trials. In the second study, participants from Ohio State University were also recruited to obtain viewpoints from an additional CCC. Eligibility criteria were otherwise the same for both studies with the exception that subjects who participated in the first study were not eligible to participate in the second. Additional details on the recruitment methods are available in the Data Supplement. Institutional review board approval was obtained before each study was initiated.

### Moderator guide development and procedures

Moderator guides were developed based on existing reviews of cancer research participation disparities, the NIMHD framework, and our prior studies of leukemia trial enrollment disparities.^6^^,^^8^ Guide structure included an introduction; objectives and ground rules; opening, content, probing, and closing questions; and a wrap-up. Detailed guide development methods and focus group procedures are available in the Data Supplement.

### Analysis

Given the studies’ objectives, we had anticipated that a minimum of 4 focus groups each would be needed to address their respective research aims.^22^ After each focus group, transcripts were reviewed, and data collection for each study was considered complete when the team determined that meaning saturation had been achieved and no substantially new perspectives were introduced.^23^^,^^24^ To synthesize the data from the 2 studies, we performed an amplified supplemental secondary qualitative analysis. *Amplified* refers to an analysis that examines common themes across datasets; *supplemental* refers to an analysis that focuses on a portion or aspect of the data, especially one that was only partially addressed by the initial data; and *secondary* refers to an analysis that is not the direct and primary analysis of a single study’s data.^25^ Such an approach was deemed appropriate given the objectives of the studies being blended in relation to the objective of the analysis; this approach was guided by framework analysis and included prefigured and emergent dimensions.

The NIMHD framework served as the foundation for the codebook used in the secondary analysis. The team then used an iterative approach to codebook development, and additional codes were added to capture concepts and ideas in the data that fell outside of the NIMHD framework. Specifically, 2 project-specific levels of influence were identified and situated alongside existing NIMHD levels in the codebook: Trial Design (eg, inclusion and exclusion criteria) and Institutional (eg, dynamics specific to the local healthcare system). Once a comprehensive codebook was finalized, the new dataset was then recoded. Codes were then summarized, reviewed, and discussed by the team to identify the patterns, concepts, and contexts associated with barriers and facilitators to clinical trial enrollment. Initial themes were developed and revised through discussion, with a focus on situating them within the relevant domains and levels of the NIMHD framework and identifying concordance and discordance within and between participant types. Coding and analysis were assisted by NVivo 14 software. Results presented below highlight barriers that were considered relevant to the unique care delivery patterns of acute leukemia and clinically significant. Additional details regarding data analysis are available in the Data Supplement.

## Results

**Figure 1.**
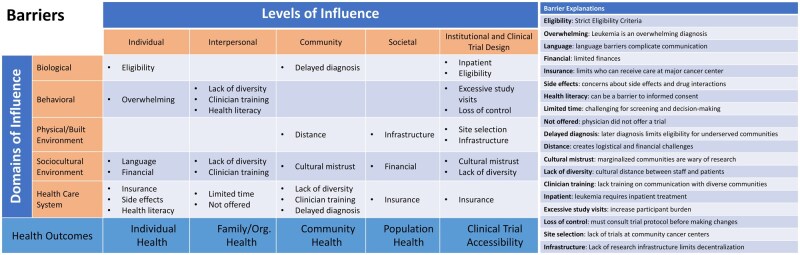
Barriers to leukemia trial enrollment equity on the modified NIMHD research framework. Barriers identified by patients and clinicians are organized according to the levels and domains of influence in the modified NIMHD research framework. Short titles are provided for each barrier, with more detailed information available on the corresponding list. NIMHD: National Institute for Minority Health and Health Disparities.

**Figure 2.**
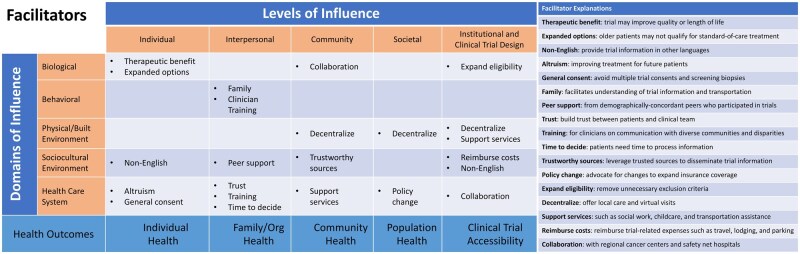
Facilitators to improve leukemia trial enrollment equity on the modified NIMHD research framework. Facilitators and solutions identified by patients and clinicians are organized according to the levels and domains of influence in the modified NIMHD research framework. Short titles are provided for each barrier, with more detailed information available on the corresponding list. NIMHD: National Institute for Minority Health and Health Disparities.

The results presented below summarize those barriers and facilitators that were considered relevant to the unique care delivery patterns of acute leukemia. Representative quotes are shown in Table 1, and alignment of key barriers and facilitators within the modified NIMHD framework is shown in Figures 1 and 2, respectively. A comprehensive reporting of all barriers and facilitators discussed by participants is available in the Data Supplement and organized by modified NIMHD levels.

**Table 1.** Representative quotes.

Level of influence	Quotes
Individual	Barriers: “Yes, I agree with the gentleman before, fear, is a big thing. Because I know when I was offered a clinical trial, that was my biggest thing. I didn’t want to be somewhat like a lab rat. I didn’t want to go through something that would fail or would make me worse than what I was going through. So fear was a really big thing. And then all those terms, reading all those documents that come along with them, it’s overwhelming. I'm like, oh, my God. Wow.” (Patient) “I think with leukemia as an illness, it is a somewhat overwhelming, thunderous, very complicated to sort of start getting your head around treatment, sometimes happening in a very sudden way. And to shift gears from the discussion about the new diagnosis and all that’s involved to the, and here’s a clinical trial with five arms that you can join. That’s a tough—I mean, it’s hard in any disease, but I think leukemia is a particularly tough disease.” (Clinician)
	Facilitators: “Well, like I said, somebody’s got to do it. You can’t get research to move forward without somebody being, for a lack of a term, guinea pig. And if there’s something out there that I can be administered that’s going to help me also, I'm all for it. In the business I used to be in, we used to do a lot of trials to improve our business. So, I don’t think it’s much different than that. In the end, it’s all a business, let’s improve ways to do things. We wouldn’t be sitting here right now if they hadn’t figured out how to do this Zoom thing, right?” (Patient) “Wouldn’t it be great if we could just consent patients and say, we’re going to collect this one extra tube from your bone marrow and that’s going to be good for any trial that you end up going into, instead of having to overwhelm them with their diagnosis, and then also consented to ten different trials so that we don’t have to repeat the bone marrow biopsy.” (Clinician)
Interpersonal	Barriers: “And the travel—and it’s just that I don’t drive, and my husband is the only one driving and he’s working. It will really take his time away from work, and he does not have a lot of vacation time. Right now he’s doing it when I need to go to Dana-Farber for my checkup; he has to take time off from work…” (Patient) “The thing is, outpatient clinical trials is that it’s a big time commitment for these patients, especially with their distance from the hospital. And that can be a big barrier to enrollment for patients because further they are away, less support that they have from family, friends, et cetera, they’re unable to meet the requirements of the trial to come to the clinic three, four days out of a seven-day week. Or if they have children that they have to take care of, they’re—these sort of things really impact whether or not we offer trials. Because part of the eligibility criteria is are they—not only are they able to sign consent, but are they able to meet the requirements? It’s a pretty big barrier, actually.” (Clinician)
	Facilitators: “And I would say, when I think about the times I’ve had to take the clinical trial, I feel like the trust I have in my oncologist is one of the reasons why I’m able to confidently say I will go ahead with it, because I know that they have—they are not looking at it from just being a scientist or being a medical doctor, they are also looking out for the best option for me to get me—to curing my disease.” (Patient) “I guess I just would imagine if you had somebody that looked like you, spoke the same language as you, you would just—it would be easier to instill comfort and trust in the things that you’re signing up for, would be my guess.” (Clinician)
Community	Barriers: “I can only speak for my community, the Hispanic community. They’re not very well-educated. So it could come from a lot of people going undiagnosed for a very long time to where they don’t even have the opportunity—it’s too late for them to even be included in these trials, just based off of being uneducated of your—of symptoms and coming to the doctor on time, which could lead to some of these demographics being underrepresented.” (Patient) “That being said, with leukemia care in the clinic, especially acute leukemia, they’re there one to two times a week. So depending on what your resources are, your ability to get to and from the clinic is incredibly difficult, and a lot of times we refer these people back to the community because some care is better than no care. So a lot of it—and it’s not even on here—is access. Like access to us. And I think the only way that’s ever really going to get fixed is if you have a mini [academic center] that goes down to [the safety-net hospital]. But it’s not possible because of the rules and regulations around clinical trials. You need a whole team. I mean, we have 20 CRCs, five, six nurse practitioners. I mean, it’s just—that’s like a big world—or a big issue. A big issue that’s really hard to fix.” (Clinician)
	Facilitators: “I have a—also have a [local satellite] in [redacted], which is a much closer commute. And so, I was eligible for shared care. And so, that made it very easy. So, although I was doing two appointments a week, they were able to split it between the two, which really wasn’t so bad.” (Patient) “For minorities, sometimes getting some of the information from maybe like another minority, like I don’t know. Think about like videos with other minorities who have gone through the trial or some other way to access some of the information maybe from a source they trust because I think it’s hard getting new diagnosis and then having maybe a physician who has to do the consent give you information. But if you could have another layer as part of the information, like patients who have gone through the trial or clinical trials in general, maybe speaking in Spanish like a video like the patient aspect, even like logistics aspect, like the studies and how—communication and that type of thing.” (Clinician)
Societal	Barriers: “I also think the wallet biopsy that hospitals in our area do for patients in regards to, can you even come here. Acute leuks can get into the [academic center]. Sometimes, we’ve had to send people back because the [academic center] doesn’t cover, but more time than not, the [academic center] doesn’t cover their insurance. So it’s difficult to talk to a patient about a trial where there’s the question of when they get out of the hospital, can they even come to the [academic center]? So I think a big issue is education and what, as a society, we’re doing about health care coverage and make people feel comfortable of all walks of life, to be able to come to a major cancer center and get treated.” (Clinician)
	Facilitators: “Yeah, you would have to—it will take big changes. But I think it’s really important, and I think that especially with what’s been going on the last couple years and how aware people have been made of these things, that change is definitely possible and the people that want to be involved are definitely there. But I think this is a really important topic to study and write about, especially in important medical journals, and kind of get everybody onboard. And then I think hopefully that trickles down into thinking of innovative ways to conduct important clinical trials.” (Clinician)
Institutional	Barriers: “I didn’t go through a clinical trial. I went through standard stem cell transplant treatment. But I remember thinking, there’s a lot of White people here. I don’t see—I don’t see other people, like other people not like—not like me. I'm Hispanic. But I'm White. But I—I was like, wow. Is this like a White-only place? I just felt like—like what they were saying, you feel—in my case, I felt kind of like, there’s a lot of White people. Where’s everybody else that this is happening to?” (Patient) “In our cancer center, we have infrastructure, but a lot of other cancer centers may not have the infrastructure to handle all the extra work involved in a clinical trial.” (Clinician)
	Facilitators: “And collaborations, like with [safety-net hospital], with clinicians at other sites are one way to—that we’ve also tried to approach that.” (Clinician)
Trial design	Barriers: “What I would also say is that certain clinical trials have such stringent inclusion criteria and exclusion criteria that certain genetic backgrounds may make it—may make it more difficult to qualify for that particular trial. And I know that if you’re looking for one particular mutation, just some of that depends on—it’s a little bit subjective in how many different things that your doctor tests you for to make sure that you qualify for everything that they want. And if you end up with a doctor that’s more rigorous or a doctor that’s less rigorous might depend on whether people get included in the trial.” (Patient) “I think screening windows are one thing, right? So if you have low-level financial resources, you come from a far geographic location, you have a low level of social support, your ability to screen within a seven-day window for a trial may rule out quite a few people because it’s multiple trips to our institution to screen. And they have things like you have to get your EKG on our machine. You have to have your marrow with us. And those things are actually pretty big barriers.” (Clinician)
	Facilitators: “I was going to say from a perspective of like what we could do to make things better, just like really trying to coordinate so that the studies can be done at times that are convenient for the patient or like if there’s an EKG that needs to be done, trying to do it at the same time really coordinated well with that because I mean, I know everyone always tries to do that, but I think that could kind of increase the success for people that maybe live farther away or maybe that person without much social support or the old person going to clinic is really challenging from a mobility perspective.” (Clinician)

### Participant characteristics

Eight focus groups and 7 individual interviews were performed with a total of 61 participants (34 patients and 27 clinicians). Table 2 shows details of the participant types and their characteristics. Patients were majority female (68%) and from historically underenrolled races and ethnicities (82%), and approximately one-third had prior experience with trial participation.

**Table 2.** Participant characteristics.

Patients (*N* = 34)			
Category	Subcategory	*n*	%/IQR
Age	Years (median)	53	45.64
Sex	Female	23	68%
	Male	9	26%
	Unknown	2	6%
Race/ethnicity	Hispanic	9	26%
	Non-Hispanic Asian	8	24%
	Non-Hispanic Black	11	32%
	Non-Hispanic White	4	12%
	Unknown	2	6%
Year of diagnosis	2021 or later	4	12%
	2020	5	15%
	2019	10	29%
	2018 or earlier	13	38%
	Unknown	2	6%
Prior clinical trial participation	Yes	11	32%
	No	20	59%
	Unknown	2	6%
**Clinicians (*N* = 27)**			
Age	Years (median)	47	32.58
Sex	Female	11	41%
	Male	16	59%
Race/ethnicity	Hispanic	2	7%
	Non-Hispanic Asian	3	11%
	Non-Hispanic Black	0	0%
	Non-Hispanic White	22	81%
Years in practice	<10	11	41%
	≥10	16	59%
Type	Physician	21	78%
	NP/PA	6	22%

### Barriers

Patients reported low awareness of clinical trials and expressed concerns about adding to an already intensive leukemia treatment course. The anxiety, time pressure, and overwhelming information associated with a new leukemia diagnosis complicated trial communication and screening. Both patients and clinicians discussed referral, transportation, and associated logistical challenges as key barriers to trial participation, particularly in the context of leukemia (due to the need for upfront inpatient treatment and/or frequent visits). Related issues included parking, food, and hotel costs; transportation logistics; and time off for caregivers, which added to financial strain. For patients receiving HSCT, there was a lack of desire to participate in additional trial treatments beyond an already intensive process. For clinicians, the need for both specialized leukemia care, the research infrastructure, and related regulatory policies complicated offering trials closer to where patients live.

The dearth of clinician and research staff diversity was also reported to undermine trust for patients from marginalized groups. Several participants highlighted cultural distance between minoritized communities and study teams. This was perceived as being exacerbated by some patient misconceptions of trials and some clinician communications lacking cultural competence. Clinicians viewed insurance coverage as a significant barrier at the societal level, with one criticizing the “wallet biopsy” to which patients were subjected to confirm coverage. This was felt to limit who could be treated or enrolled at a CCC.

Trial design factors, including restrictive eligibility criteria, site selection and the absence of decentralization, and time-limited screening windows, also were viewed as barriers. Both patients and clinicians noted that stringent inclusion/exclusion criteria often served as a barrier. Several patients asserted that they had volunteered but were deemed ineligible for all available trials. Clinicians reported that excessive study visits and tests were barriers to enrollment for patients from marginalized backgrounds due to time commitment and logistical challenges. These factors, combined with a time-limited screening window, ruled out patients with fewer resources or who lived further from the CCC.

### Facilitators

The potential for therapeutic benefit and advancing treatment for future patients motivated patients to consider trial participation. Good communication in layman’s terms from a trusted source was seen as vital to allaying fears. To address the barrier of patients feeling overwhelmed during the inpatient, peri-diagnostic period—which was reported to be exacerbated by consenting patients for multiple trials to avoid multiple biopsies—one clinician suggested general consent for trial screening through which multiple bone marrow samples could be used for whichever trial(s) the patient might be eligible for and pursue. Clinicians also noted that the existing lack of specific leukemia therapies for older adults, in combination with the increase in experimental targeted agents, meant that these patients more frequently had trial-based treatment options.

Patients were concerned that clinicians may not recognize biases in which patients were being approached or enrolled, and they suggested that physicians be made aware of disparities in their enrollment, trained to have discussions with underrepresented groups, and motivated to discuss trials. Clinicians proposed using trusted sources of information for minoritized communities to disseminate culturally sensitive information about clinical trials. The importance of social networks, including family and peer support, was extensively discussed. Patients relied on family for trial informational and logistical support. Several examples of family facilitation were discussed such as being a “medical translator” and dispelling myths about trials. Many patients discussed their reliance on family for transportation, which became more complex with trials and would be allayed by cost coverage. Patients also considered demographically concordant peer support as potentially helpful for understanding trial participation and experiences.

Patients and physicians stressed the importance of logistical and financial support to alleviate the burdens of travel for frequent study visits. Despite barriers, efforts to decentralize leukemia trial care were seen by patients and clinicians as vital to improving enrollment equity. Suggestions included offering local study visits and structured referring and referral site partnerships between physicians and care navigators, who could also aid in identifying trials for patients diagnosed outside the CCC. Clinicians endorsed advocating for insurance policy changes to facilitate more equitable access to trials and care at major cancer centers.

## Discussion

In this qualitative analysis, we characterized barriers and facilitators specific to acute leukemia clinical trial enrollment equity at CCCs that span multiple levels and domains of influence in the NIMHD research framework. Lack of awareness and quality information about trials, a compressed time window for decision-making and screening, strict eligibility criteria, logistical challenges of referral and travel to a CCC, and inadequate insurance were key barriers to leukemia trial participation. Facilitators and potential solutions included using trusted information sources (eg, community members) to reach minoritized communities, general consent for trial screening, loosening or removing unnecessary exclusion criteria, decentralizing trial-related care, travel assistance, and expanding time and monetary cost coverage. Notably, several idiosyncratic aspects of acute leukemia care—inpatient treatment, limited time between diagnosis and treatment initiation, HSCT, and the high prevalence of quaternary care—presented unique barriers or complicated the implementation of solutions. Taken together, these factors explain what makes achieving enrollment equity distinctly challenging for leukemia trials and offer potential strategies to intervene.

Centralized care presents a significant challenge for acute leukemia enrollment equity. Participants repeatedly highlighted logistical and travel-related barriers associated with trial participation, which are offered at substantially higher rates at CCCs.^20^ Factors driving trial centralization and these related barriers include its relatively low prevalence, the need for inpatient treatment and frequent outpatient visits, and the fact that some trials involve HSCT, which requires further specialization. Experimental therapy and post-HSCT care also necessitate frequent monitoring by specialists, though notably our “shared care” HSCT model between specialists and local sites has been shown to be effective and might be applied to the leukemia trials setting.^26^ Still, HSCT candidacy usually requires patients to identify a dedicated caregiver to facilitate safe post-HSCT care.^27^ This introduces an additional barrier, and research has shown that adherence to HSCT requirements is more challenging for lower socioeconomic status families and results in lower receipt of HSCT.^28^

Despite these challenges, there was a clear consensus that cancer centers conducting leukemia trials should aim to decentralize where possible. Trialists should consider collaboration with sites that may serve more underrepresented populations. Standard-of-care procedures such as lab draws and transfusions should be able to be provided locally; policy is evolving to remove existing regulatory obstacles for such practices.^29^ When offering trial visits closer to home is not possible, financial and logistical support may be crucial to overcoming barriers to enrollment equity. The Food and Drug Administration (FDA) had been encouraging such strategies through legislation that required pivotal trials to submit Diversity Action Plans outlining anticipated barriers to diversity and plans to overcome them, but these are currently not being enforced.^29^^,^^30^ Advancing enrollment equity might be best achieved if sponsors and cancer centers take on more of the logistical and financial burdens of trial participation, which currently fall on patients and their families. For example, reimbursing travel-related expenses has been shown to significantly diminish cost concerns related to travel and lodging.^31^ Moreover, pragmatic trial designs are often more feasible to open at community practices and less burdensome for patient participants.^32^ These trials offer greater flexibility during treatment and broader eligibility criteria, which may improve enrollment of underrepresented groups and increase generalizability.^33^

In addition to specialized care access, acute leukemia diagnosis and treatment often occur in the inpatient setting. This is a challenging environment for informed consent, particularly given that treatment initiation quickly follows diagnosis. Participants noted that time delays caused by informed consent and screening for trials heighten an already anxious peri-diagnostic period. While these factors should theoretically affect all patients, suggestions from participants to improve enrollment equity through clearer communication and building trusting relationships are particularly challenging to implement in this context. Offering more time to process information, while important for ideal decision-making, conflicts with the need to provide timely treatment. While an acute leukemia diagnosis is often seen as an emergent situation requiring immediate treatment, a recent systematic review and meta-analysis found conflicting evidence. Data suggest it may be safe to delay treatment initiation for additional testing if patients are clinically stable^34–36^; this would provide more time to process clinical trial information and complete necessary screening. Umbrella trial design can match patients with experimental therapies based on their genomic data; for example, an umbrella trial of precision medicine treatment for acute myeloid leukemia (AML) found that genetic testing was feasible within 7 days without impacting overall survival.^35^ This aligns with the suggestion from clinician participants to avoid duplicate biopsies by using samples to screen for multiple trials.

Participants also identified barriers relevant for trials agnostic of cancer type. Informed consent is known to be complicated by patient anxiety.^37^^,^^38^ Limited health literacy and language barriers are also associated with reduced trial participation.^39^ Participants reported low knowledge about trials, suggesting a need for rigorously tested and culturally sensitive delivery of trial information. The American Society of Clinical Oncology (ASCO) released a research statement recommending communication training for researchers and collaboration with community leaders to disseminate trial information.^40^ Peer support was discussed by participants as a way to improve understanding and trust of clinical trials. While potentially beneficial, peer influence also may be misleading, as patient experience and clinical trajectory for acute leukemias are highly individualized. Although mistrust among minoritized patients is often cited as a reason for their under-enrollment, a systematic review found no difference in enrollment rates based on race or ethnicity. The authors urge a greater focus on eligibility criteria and systemic barriers to improve enrollment.^15^

Strict eligibility criteria have also been shown to limit blood cancer enrollment equity.^41^^,^^42^ Indeed, experiences shared by patients and clinicians in this study regarding overly strict eligibility criteria are supported by our recent analysis of phase II-III acute leukemia trials, in which we found eligibility criteria to be more restrictive than justified by drug safety data.^43^ These findings reflect a broader trend in cancer trials, prompting ASCO and the FDA to advocate for increased justification of eligibility restrictions.^44–46^ For example, common exclusion criteria such as diabetes, hypertension, and hepatitis status disproportionately exclude marginalized populations.^47^ While safety is paramount, unjustified eligibility criteria undermine the generalizability of results and perpetuate inequitable trial access.

Finally, our data suggest that additional changes may be needed at the societal level. Disparities in trial access reflect broader inequities in US healthcare. As a clinician in this study recognized in their comment about the “wallet biopsy,” patients with inadequate health insurance can be systematically excluded from both high-quality care and research participation at some major cancer centers. Changes to health insurance coverage can address this barrier. For example, 2 recent analyses found that Medicaid expansion improved cancer trial enrollment for those patients^48^ and increased racial/ethnic diversity of cancer trial participants in states where coverage of trial participation by Medicaid is mandated.^49^ Advocating for systemic change is important to not only remove barriers to equity of trial enrollment, but also to ensure that the benefits of cancer research are shared by all communities.

This study has several limitations. The sites involved may have biased the sample toward patients with some degree of access to academic centers or referral sites. Notably, however, trial access was a primary concern even among these patients. A community physician perspective would have provided additional insights as to how decentralized trials may be performed and barriers to referral in the peri-diagnostic period; this perspective should be further explored. We were able to identify which patient participants had taken part in a clinical trial in the past (shown in Table 2) but did not assess who had been offered to participate but ultimately declined or was ineligible. Although a diverse sample of patients participated, clinicians were primarily non-Hispanic White (81%), which reflects the makeup of the oncology workforce and itself has been linked with inequities in research participation and was noted by patient participants.^50^ Finally, by virtue of being a secondary analysis, this analysis combines results of 2 studies with related, but distinct aims which can add hidden and potentially interactive sources of bias. At the same time, synthesizing these data allowed us to perform a rich, in-depth analysis of a substantial sample of patient and clinician stakeholders.

## Conclusion

This characterization of barriers to equity in acute leukemia clinical trial enrollment and potential solutions represents a key step toward improving representative enrollment. These data point to targets for complementary quantitative research and future interventions across multiple levels of the NIMHD research framework. Notably, actionable barriers were found at the individual, interpersonal, community, societal, institutional, and trial design levels. These findings suggest that a multi-pronged approach is needed to remove barriers to enrollment and ensure all communities have the opportunity to contribute to and benefit from acute leukemia clinical trials.

## Supplementary Material

oyaf241_Supplementary_Data

## Data Availability

The data underlying this article cannot be shared publicly due to institutional review board restrictions on data sharing. The data will be shared on reasonable request to the corresponding author contingent on approval from the institutional review boards who approved this research.
