# Supplementary material for: Barriers and facilitators to representative acute leukemia trial participation at National Cancer Institute-designated comprehensive cancer centers
Source: Oncologist. 2025 Aug 8;30(9):oyaf241. doi: 10.1093/oncolo/oyaf241 (PMC12445654; doi:10.1093/oncolo/oyaf241)
Supplement: oyaf241_Supplementary_Data [file oyaf241_supplementary_data.zip › Data Supplement the oncologist.docx]

**Data Supplement**

1. Recruitment
2. Guide Development
3. Focus Group and Interview Procedures
4. Study 1 Patient Moderator Guide Questions
5. Study 1 Clinician Moderator Guide Questions
6. Study 2 Patient Moderator Guide Questions
7. Study 2 Clinician Moderator Guide Questions
8. Detailed Barriers
9. Detailed Facilitators

Recruitment

Patient participants were identified and screened using institutional cancer registries. Clinicians were identified and screened using departmental directories. Patients were recruited using mailed letters with phone and letter follow-up for non-responders, and clinicians by email with email and phone follow-up. Purposeful sampling approaches were used to maximize demographic diversity among both groups by age, sex, race, ethnicity, and socioeconomic status, as well as practice type and years of experience for clinicians.

Guide Development

Introductory paragraphs, oral consent scripts, and portions of the guides that discuss other subjects and are thus not relevant to the present analysis have been removed. Questions sought to enable an open-ended multi-level (patient, clinician, trial, institution, societal) exploration of domains and levels through their application to the participants such as their familiarity and/or experience with clinical trials, factors influencing their participation or approaching patients for participation, participant and clinician concerns and enrollment supports, and facilitators to address enrollment barriers and disparities. Each guide was iteratively revised by the research team and pilot tested with two individuals.

Focus Group and Interview Procedures

Focus groups were approximately 90-minutes long and interviews were between 30-45 minutes long. Both used video conferencing software with a phone-in option. Focus groups were held separately by participant type (patients and leukemia clinicians). Sessions were audio recorded and professionally transcribed. Focus groups were moderated by PhD-trained qualitative methodologist (AR and JER). Oral informed consent was obtained from participants immediately prior to focus group or interview participation.

**Study 1 Patient Moderator Guide Questions**

1. What do you think of when you hear the term clinical trial?
   1. How familiar or unfamiliar are you with clinical trials as a part of leukemia treatment?

To clarify this term for the purposes of today’s discussion, a clinical trial is “a research study that tests the use of a medication or medications in order to find better ways to treat patients with particular diseases.” Clinical trials include testing a new medication or testing new ways to deliver existing medications.

- 1. Do you have any initial reactions to this definition?
  2. From your perspective, what might be some positive things about clinical trials, if any?
  3. What might be some negative things about clinical trials, if any?
  4. What questions do you have about clinical trials?

1. Here is the definition of a clinical trial that we showed you before. (show definition on screen) Has your leukemia care team ever discussed participating in a clinical trial with you?
   1. If not discussed 🡪 to what extent would clinical trials be something you are or are not interested in participating in?
      1. Can you walk me through some of the reasons that lead you to feel this way?
         1. What are some reasons you might consider clinical trial participation?
         2. What are some reasons you might avoid clinical trial participation?
         3. What questions would you have if it was offered to you as an option?
         4. If not interested 🡪 Why not?
            1. On the screen you will see a list of concerns that we have heard or read about other patients having. I’d like to get a better sense of which, if any, are applicable to your situation: (share screen)

The feeling of being “experimented on”?

Cost of participation / insurance coverage

Travel costs and/or transportation

Time in the hospital

Side effects

Trust in your healthcare team

Trust in the medical system

Privacy (e.g., your blood or bone marrow samples being used for things you did not consent to)

- - - - 1. What, if anything, could be done to address or lesson some of these concerns?
        2. (if time ask if they would add anything to reflect their experience)
      1. If yes interested 🡪 Did you ever try to discuss research studies with your healthcare team?
  1. If yes discussed 🡪 when you considered participating in a clinical trial, which factors were most important for you when deciding to participate or not?
     1. Since these previous situations, how have your concerns change, if at all, over time?
     2. Did you end up participating in any clinical trials?
        1. If not participating 🡪 why not?
           1. Probe on above concerns to see if they were important factors.
           2. Can you think of anything that could have be done to address or lesson these concerns?
        2. If yes participating 🡪 How was that experience for you personally?
           1. Can you describe any of the concerns you had about participating that ended up impacting your care?
           2. What was done to address these concerns or reduce their impact, if anything?
           3. Were there any supports or resources that you think would have been helpful?

What additional measures could have been taken to reduce the impact of these problems?

1. One of our goals is to better support patients as they consider participating in clinical trials … , thinking about all of the potential concerns and barriers that patients can face:
   1. What do you think can be done differently to alleviate/lesson patients concerns about participating in clinical trials?
      1. If you have been in the hospital when your doctor discussed a study with you, did your being in the hospital change what you thought about participating or did it not make a difference?
   2. Are there any specific resources or supports that would be most helpful?
2. Prior studies suggest that clinical trials … disparities exist along racial, gender, age, and economic lines. Meaning that for patients from diverse racial and ethnic groups, women, older adults, and those with less financial resources, there are lower rates of participation in clinical trials.
   1. What is your initial reaction to hearing about this disparity?
3. Recently, the medical community has also been concerned that non-medical factors such as a person’s sex, age, race, gender, ethnicity, or financial status may be impacting how often they are offered participation in clinical trials.
   1. What is your initial reaction to hearing this?
   2. What are your thoughts on how, if at all, non-medical factors influence a medical team’s decision to offer a clinical trial … to patients?
      1. Do any examples come to mind?
   3. What, from your perspective, is the most appropriate way to respond to this issue?
   4. Thinking about the concerns and barriers we have already discussed, what types of information or support would be most meaningful to increase participation for these under-enrolled groups?
4. Are there any additional topics that you’d like to address or anything else you’d like to add before we finish up today?

**Study 1 Clinician Moderator Guide Questions**

1. I’d like to start broadly by asking what you see as the major barriers for patients looking to enroll on to a clinical trial. What about non-patient barriers. Probes:
   1. Patient inclusion or exclusion criteria?
      1. Organ function
   2. Trial protocol restrictions?
      1. Number of visits
      2. Inpatient treatment
      3. Inability to receive local care –
   3. Placebo controlled trials
   4. Trial complexity?
   5. Consent form complexity?
   6. Lack of staff?
   7. Availability of trials for those at community hospitals?
      1. Community outreach
      2. Community hospital partnerships
   8. Cost of trial participation?
      1. Time
      2. Loss of work
   9. Patient hesitancy?
      1. Beliefs / fears misconceptions
   10. Rapidity from diagnosis to treatment?
       1. Ability to temporize
   11. What measures are available to you for overcoming … barrier?
   12. What else do you think can / should be done to overcome that barrier?
   13. Of the barriers we’ve discussed, which do you think are the most important nationally? Locally?
       1. Which do you think are the easiest to address?
2. Are there barriers that impact … more than others? Probes:
   - 1. Older adults
     2. Females
     3. Non-English speaking persons
     4. Patients who live far from your institution
     5. Those with less income
     6. Those with less social support
     7. Those with more restrictive insurance payors
     8. Health literacy
     9. African American/Black persons
     10. Hispanic Americans
     11. Asian Americans/Pacific Islanders
3. What measures are available to you for tracking how your trial enrollees compare to your institution’s patient population? (Explicit definition: how your aggregate enrollment and trial eligibility compare to who is seen and able to enroll)
   - 1. What about how your enrollee population tracks against the cancer center’s catchment?
   1. What, if any, enrollment statistics would be helpful to you at the provider or institutional level?
      1. How might you want to see these data?
4. What measures, if any, are being taken to expand acute leukemia enrollment to clinical trials?
   1. What further measures would you like to see be taken, if any?
   2. Any measures focused on patients?
      1. What do you think about timely, demographically concordant peer support?
   3. Any measures focused on providers?
      1. At institutions with trials?
      2. At referring institutions?
   4. Any measures focused on the trial itself?
   5. Any measures focused on institution?
5. Are there any additional topics that you’d like to address or anything else you’d like to add before we finish up today?

**Study 2 Patient Moderator Guide Questions**

Before we begin the discussion, I’d like to define what we mean when we talk about a clinical trial … .

For the purposes of this discussion, a clinical trial is a “research study that people diagnosed with leukemia volunteer to take part in, and it tests how well a treatment for their leukemia works. These trials can be for people who were recently diagnosed and have never received treatment before, or for people who have received other treatments. There are other types of trials and research, but for today, we will just be talking about the kind that tests how well a treatment works.”

- - - 1. First, I’d like to hear your initial reaction to hearing that clinical trial participants are not representative of all people diagnosed with leukemia, meaning that there are some groups of people with leukemia who participate in clinical trials less than others.
         1. What are some reasons you think this might be the case?
      2. What impact do you see this having on improving the treatment of leukemia?
         1. Can you describe any positives or negatives about the lack of diversity in leukemia clinical trials?
      3. What are some concerns people might have about participating in a clinical trial?
      4. What … [are] ways can we encourage doctors to improve the representativeness of their trials?
      5. What … supports or tools, if any, should the researchers pursue to improve the representativeness of clinical trials?
      6. Are there any additional topics that you’d like to address or anything else you’d like to add?

**Study 2 Clinician Moderator Guide Questions**

1. What are the main issues you face in trying to recruit under-enrolled populations, such as those from race-ethnic minority backgrounds, to your trials?
2. What about multicenter trials where you work with other institutions?
3. What do you and your group do to improve the racial and/or ethnic diversity of enrollees at your site?
4. What about multicenter trials where you work with other institutions?
5. How do you assess equitable enrollment in clinical blood cancer research?
   1. Probe about trial recruitment if not addressed directly.
6. How useful, if at all, do you think audit and feedback about equitable care is for your research?
   1. How about for your practice?
7. What … interventions, if any, should the researchers pursue to improve the representativeness of clinical trials?

Detailed Barriers

*Individual Level*

Patients and clinicians identified several individual barriers to clinical trial enrollment that were specific to or more challenging because of the natural history and treatment cadence of acute leukemia. Patients reported that the information about their diagnosis and trials were often provided in non-ideal ways (e.g., only through verbal discussions with or without text print-outs, using unfamiliar medical terms), which exacerbated the already anxious peri-diagnostic period. The compressed timeline from diagnosis to treatment also constrained their desire to commit to trial participation. For patients receiving HSCT, there was a lack of desire to participate in additional trial treatments beyond an already intensive process. Clinicians’ comments were aligned; they also reported that the potential for treatment delays due to trial decision-making and screening decreased some patients’ interest in trial participation. Leukemia-agnostic barriers were also discussed. These included concerns about side effects, unknown interactions with current treatments or non-oncology medicines, the need for financial means to attend extra trial-related appointments, high health literacy, English proficiency, and the related experimental nature of the trial, even if not placebo-controlled. There was also worry from patients that participating may make them ineligible for a future approved treatment. Patients expressed a general lack of awareness and knowledge of trials (e.g., if and when they are available, what they entail, or how to enroll). For patients previously offered participation, they noted that information was often confusing and overwhelming. Several assumed that trials were only used for “last ditch efforts.”

*Interpersonal Level*

Peri-diagnostic anxiety related to the rapidity of diagnosis and treatment was discussed as creating a challenging environment for medical decision-making. Notably, six patients stated that their oncologists had never discussed clinical trials with them and assumed that trials were not an option for them because their doctor did not offer it.

*Community Level*

Several highlighted cultural distance between minoritized communities and study teams. This was perceived as being exacerbated by some patient misconceptions of trials and some clinician communications lacking cultural competence. Another noted that their Hispanic community tended to have delayed medical diagnoses that made them less eligible for clinical trials. Clinicians noted that physical distance also made timely trial screening challenging, especially for patients with limited financial resources who could not get off work. Lack of trial availability at community hospitals exacerbated these challenges.

*Societal Level*

Clinicians viewed insurance coverage as a significant barrier, with one criticizing the “wallet biopsy” to which patients were subjected to confirm coverage. This was felt to limit who could be treated at a major cancer center and be enrolled on trials. Clinicians mentioned the barrier of unexpected costs associated with being on a trial. Some patients agreed with this concern while others noted that their insurance covered all costs while they participated. Of specific concern were costs associated with trial logistics, which are discussed further in Trial Design below.

*Institutional Level*

Several patients said they were not able to access trials because of where they received initial care. Due to the rarity of acute leukemia and the need for expertise, they reported that trials were not offered locally; clinicians echoed this, stating that trial screening or participation by community hospitals was not feasible due to the lack of research infrastructure necessary to supportive intensive leukemia trials. One patient participant described being open to trial participation if it were available closer to home but not if it required being treated exclusively at the academic center. Academic centers were also viewed as “white” places, that is, patients who self-identified from minoritized racial and ethnic groups felt like there were few others who reflected their demographics. Throughout the course of treatment, however, they reported seeing more patients and staff that were “like them,” which they found reassuring.

*Trial Design Level*

Community level barriers discussed above relating to site selection also relate to trial design. Lack of trial availability at community hospitals was seen as a significant barrier to enrollment equity. Additionally, both patients and clinicians noted that stringent inclusion/exclusion criteria often served as a barrier. Several patients asserted that they had volunteered but were deemed ineligible for available trials. Earlier phase trials also gave some patients pause due to concerns about lack of safety data. Clinicians reported that excessive study visits and tests were barriers to enrollment for patients from marginalized backgrounds. These factors, combined with a time-limited screening window, ruled out patients with low resources or who lived too far away. Another clinician felt that randomization was challenging in the context of cultural mistrust. One clinician noted that they experienced a loss of control with patients on trials because they need to consult the study protocol before doing anything.

Detailed Facilitators

*Individual Level*

Patients had a general expectation that trial-based treatment may cure or improve their disease and extend their life, which facilitated interest in trial participation. Patients reported that some concerns about participating were mitigated when clinicians were explicit about potential reasons for participation including personal benefit as well as advancing treatment for future patients. Desired information included trial safety, expected toxicity compared to standard treatment, and interactions with current medicines. To address the barrier of patients feeling overwhelmed—which was reported to be exacerbated by consenting patients for multiple trials to avoid multiple biopsies—one clinician suggested general consent for trial screening through which multiple bone marrow samples could be used for whichever trial(s) the patient might be eligible for and pursue. Clinicians also noted that the existing lack of specific leukemia therapies for older adults, in combination with the increase in experimental targeted agents, meant that these patients more frequently had trial-based treatment options.

*Interpersonal Level*

Patients emphasized the importance of using simple, non-technical words in their preferred language when discussing trials. There was related discussion of clinicians not using paternalistic language (e.g., “I know how you feel”) with patients from marginalized groups and be open if they did not have experience treating patients from similar demographic backgrounds. Other facilitators of trial information included discussions with non-physician research staff and written guides that they could read and process at their own pace. There was an emphasis on how discussions and information mitigated fears, concerns, and uncertainty. Encouragement, optimism, honesty, and sincerity (without being pressured) about the potential benefits of trials were also seen as allaying concerns. Patients and clinicians noted that trust in the care team was critical for patient willingness to engage in trial discussions. With this trust, patients described being open to recommendations such as trial participation. Finally, patients were concerned that clinicians may not recognize biases in which patients were being approached or enrolled, and they suggested that physicians be made aware of disparities in their enrollment, trained to have discussions with underrepresented groups, and motivated to discuss trials.

The importance of social networks, including family and peer support, was extensively discussed. Patients relied on family for trial informational and logistical support. Several examples of family facilitation were discussed such as being a “medical translator” and dispelling myths about trials. Many patients discussed their reliance on family for transportation, which became more complex with trials, and would be allayed by cost coverage. Patients also considered demographically-concordant peer support as potentially helpful for understanding trial participation and experiences. Some patients noted that peers should be purposefully selected and trained so they are objective about their trial experiences.

*Community Level*

Patient facilitators focused on the social network aspects described above as well as general and trial-specific community engagement activities by the health system and/or care team. For clinicians, using information sources viewed as trustworthy by minority groups, and providing the information in their own language, were seen as potential facilitators for increasing diversity. For example, one clinician suggested creating informational videos featuring minorities who had participated in clinical trials.

*Societal Level*

Clinicians felt that policy changes were needed to fix insurance coverage and trial access barriers. One suggested that publications in major medical journals and oncology societies should continue to assess and advocate for these structural and societal changes. Specific comments as to what this would entail include policies that mandate enhanced communication, as is discussed in the Interpersonal Level above, and for the issues relating to trial conduct, as discussed below.

*Institutional Level*

Patients stated that any ability to receive trial care at local sites within the health system, such as at satellites, made them more likely to participate. They also discussed institutional resources as facilitating participation (e.g., social work, free parking, childcare, reiki therapy, and quality care across disciplines). Facilitators identified by clinicians included admitting a patient to the hospital to avoid multiple trips, involving social work, and offering virtual visits when allowed by the trial and institution. One clinician commented that despite the logistical barriers, frequent visits could be comforting to patients who wanted to be watched closely.

*Trial Design Level*

Decentralized trial care, as discussed above, was also applicable here. Patients suggested that additional financial support and cost coverage within a trial is critical. One clinician reported they had been successful at getting insurance coverage for adolescent and young adult patients, which led to improved diversity for trials in that age range. Several clinicians found collaboration with regional cancer centers and safety net hospitals facilitated diverse participation, though they noted that this could be difficult for lower volume studies. Clinicians who wrote protocols also described pushing for local therapy and travel support in their studies whenever possible.
